# Metabolic engineering of *Escherichia coli* BW25113 for the production of Vitamin K_2_ based on CRISPR/Cas9 mediated gene knockout and metabolic pathway modification

**DOI:** 10.1186/s13036-025-00614-9

**Published:** 2026-01-08

**Authors:** Changchuan Ye, Yan Zhang, Jie Zhang, Menglei Shi, Feixue Nie, Qinghua Liu

**Affiliations:** Fujian Provincial University Engineering Research Center for Animal Breeding and Sustainable Production, Department of Animal Science, College of Animal Science, Fujian Agriculture and Forestry University, Fuzhou, 350002 China

**Keywords:** *E. coli*, Vitamin K_2_, Menaquinol-8, CRISPR/Cas9, Metabolic engineering

## Abstract

**Background:**

Vitamin K_2_ (VK_2_), as a derivative of the menaquinone family, plays an important role in the prevention of osteoporosis and cardiovascular calcification. The realization of the industrialization of VK_2_ and the reduction of its production cost have become the focus of attention.

**Results:**

In this work, an *E. coli* strain with high VK_2_ accumulation was constructed through rational metabolic engineering and stepwise improvement based on regulatory metabolic information and CRISPR/Cas9-mediated gene knockout. We first constructed a recombinant *E. coli* strain BW-T7/MU to produce menaquinol-8 (MKH_2_-8, a reduced form of VK_2_) by overexpressing *menA* and *ubiE* genes, which encoding the rate-limiting enzymes of the menaquinol pathway. After 24 h and 48 h of fermentation, this strain BW-T7/MU reach a titer of 303 mg/L and 232 mg/L. Secondly, we overexpressed different related genes *wrbA* (oxidative stress mitigation), *qorB* (reduction of quinones) and *menF* (conversion of chorismate to isochorismate), respectively. Among these recombinant strains, the strain BW-T7/MUW (overexpressing *menA*, *ubiE* and *wrbA* genes) reached the highest titer of VK_2_ after 48 h of fermentation. The optimization of the medium led to an increase in the accumulation of VK_2_. Subsequently, the rational metabolic engineering of gene knockout further increased the titer of VK_2_. The recombinant strain ΔB/MUW was selected as the dominant strain for further optimization, with a high VK_2_ titer of 724 mg/L. A final attempt is to overexpress *ispB* gene to increased flux of isoprenoid side chain synthesis, resulting in strain ΔB/MUWI with a titer of 859 mg/L in a shake flask and 1360 mg/L in a 5 L fermenter after 48 h cultivation.

**Conclusions:**

The stepwise engineering strategy raised the VK_2_ titer from the initial 303 mg/L to 859 mg/L through rational pathway modification and systematic gene expression. Further optimization in batch fermentation increased the VK_2_ titer to 1360 mg/L, which highlights the strong engineering impact of our strategy.

**Supplementary Information:**

The online version contains supplementary material available at 10.1186/s13036-025-00614-9.

## Introduction

Over the past two decades, synthetic biology has evolved from foundational research to transformative applications, marked by significant advancements in metabolic engineering, gene editing, and systems biology [1]. These developments have expanded the scope of synthetic biology, enabling the design of engineered microorganisms for high-yield biofuel production, sustainable chemical synthesis, and novel material fabrication. As one of the most widely used prokaryotic systems for synthetic biology, *Escherichia coli* has been extensively studied at both fundamental and applied levels due to its rapid growth rate, well-characterized genetic background, and ease of manipulation [2]. Moreover, *E. coli* is often used as a model bacterium to define microbial cell factories for many products and to study regulatory mechanisms [3], making it the most popular expression platform. The rapid evolution of genetic engineering and synthetic biology has substantially expanded the genetic toolkit for *E. coli*, enabling precise genome editing and systematic metabolic engineering to biosynthesize diverse high-value endogenous and heterologous compounds such as our target compound, Vitamin K_2_.

Vitamin K_2_ (VK_2_), as an essential nutrient with multiple physiological functions, has been widely confirmed to play a key role in bone metabolism protection, prevention of neurodegenerative diseases, regulation of antioxidant activity, and maintenance of coagulation function. With the continuous advancement of research, the application value of VK_2_ in the field of nutritional supplementation has become increasingly prominent, and its potential contribution to promoting overall health and reducing the risk of chronic diseases is gradually becoming a focal point of scientific investigation [4, 5]. Menaquinone-8 (MK-8), as an important form of VK_2_, exhibits a wide range of biological functions in the human body, including reducing bone loss and the risk of fractures [6], and alleviating the pathological progression of neurodegenerative diseases such as Alzheimer’s disease and Parkinson’s disease [7, 8]. In addition, MK-8 also plays an indispensable role in the blood coagulation process [9]. Based on the aforementioned functions, VK_2_, as an important nutritional supplement, is receiving increasing attention and emphasis in human diet and health management.

Currently, most research on microbial production of VK_2_ focuses on *Bacillus subtilis* and *Escherichia coli*. In *B. subtilis*, the addition of surfactants markedly increased membrane permeability and electron‑transport chain activity, raising the MK‑7 titer to 59 mg/L—a gain of approximately 80% [10]. Deletion or multiplex mutagenesis of the *sinR* gene promoted the formation of a denser biofilm, achieving a maximum MK-7 titer of 100 mg/L, which corresponds to a 2.8-fold increase over the parental strain [11]. Moreover, heterologous introduction and fusion expression of the *dxs* gene enabled combinatorial metabolic engineering of *B. subtilis*, resulting in a strain that produced 474 mg/L MK‑7 in a 50 L fermenter [12]. By modifying the isopentenyl diphosphate (IPP) metabolic pathway, researchers have successfully enhanced the biosynthesis efficiency of terpenoids in *B. subtilis*, achieving an MK-7 titer of 1.55 g/L in a 50 L fermenter [13]. In *E. coli*, metabolic‑pathway engineering coupled the supply of the mevalonate precursor with a complete *men* gene cluster and overexpressed key genes such as *menA* and *ubiE* to overcome competition from ubiquinone. In fed‑batch fermentation, this strategy achieved a VK₂ titer of 1.35 g/L with a productivity of 0.03 g/L/h [14]. These studies provide valuable strategies for further increasing VK_2_ titer through synthetic biology.

**Fig. 1:**
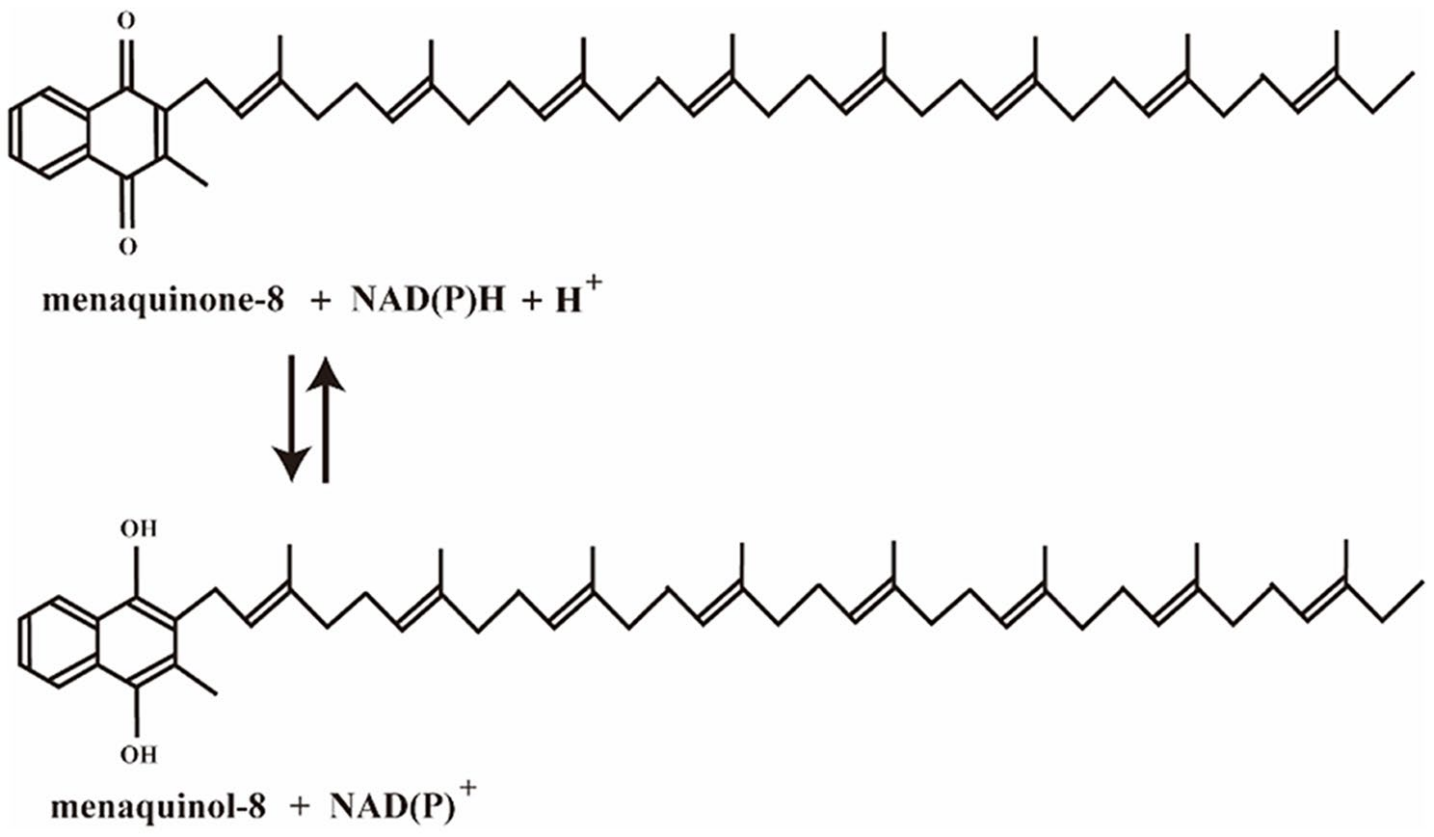
Menaquinone-8 (MK-8) and its reduced form menaquinol-8 (MKH_2_-8)

Base on the mature *E. coli* expression platform in our laboratory, we selected *E. coli* as the host for VK_2_ biosynthesis in this study. Under aerobic conditions, *E. coli* primarily synthesizes ubiquinol-8 and a small amount of MK-8, while under anaerobic conditions, it mainly synthesizes MK-8 [15, 16]. The synthesis of VK_2_ by *E. coli* mainly involves four modules: the Embden-Meyerhof-Parnas pathway (EMP), the hexose monophosphate pathway (HMP), the mevalonate pathway (MVA), and the menaquinone synthesis pathway (MK). In *E. coli*, menaquinol‑8 (MKH_2_‑8, the reduced form of MK‑8, as shown in Fig. 1) can be synthesized directly through the key gene *ubiE*, making its efficient accumulation easier. Compared with MK-8, MKH_2_-8 possesses a more chemically stable structure. Besides, intracellular metabolic pathways can be engineered to enrich MKH_2_-8 in the cell membrane, thereby improving extraction efficiency, reducing product loss and lowering production costs. Considering of these, we chose MKH_2_-8 as the final product for this study. In our study, under aerobic conditions, we modified and optimized the key MK pathway involved in the biosynthesis of VK_2_ in *E. coli* to enhance VK_2_ production by increasing the accumulation of MKH_2_-8 and reducing unnecessary metabolic flux.

In previous studies, we developed a strain capable of efficiently expressing proteins using the T7 system, named BW25113-T7. This strain has been demonstrated to express proteins efficiently using the T7 expression system with glucose as the substrate [17–19]. The T7 expression system enables higher flux‑control precision and greater scalability. We hypothesize that this strategy will enable more efficient VK₂ production under aerobic conditions. In this study, our objective is to engineer *E. coli* at the system level through metabolic engineering to generate MKH_2_-8 overproducers with fully defined genotypes. Our study employs rational metabolic engineering based on known regulatory and metabolic information to develop an *E. coli* strain capable of efficient VK_2_ accumulation.

## Materials and methods

### *E. coli* strain

Molecular cloning and plasmid manipulations were performed using *Escherichia coli* DH5α (TransGen, Beijing). BW25113-T7 strain was used for CRISPR/Cas9-induced double-strand break and recombination. All *E. coli* strains were routinely cultured in standard LB medium unless otherwise stated. Table 1 showed all *E. coli* strains and plasmids used in this study.

**Table 1:** Strains and plasmids employed in this study

Strain or plasmid	Relevant characteristic(s)	Source and/or reference
Strains		
*E. coli* DH5α	*F-*,*φ80dlacZ ΔM15*,*Δ(lacZYA -argF)U169*,* deoR*,* recA1*,* endA1*,*hsdR17 (rK-*,* mK +)*,* phoA*,* supE44*,* λ-*,* thi-1*,* gyrA96*,* rel*	Laboratory stock
*E. coli* BW25113	*F-*,*DE(araD-araB)567*,*lacZ4787(del)::rrnB-3*,*LAM-*,*rph-1*,*DE(rhaD-rhaB)568*,* hsdR514*	Laboratory stock
*E. coli* BW25113-T7	BW25113 i*nt::(lacI::PlacUV5::T7 gene) ΔybhC*	Laboratory stock
*E. coli* ΔB	BW25113-T7 *ΔentB*	This study
*E. coli* ΔC	BW25113-T7 *ΔpabC*	This study
*E. coli* ΔBC	BW25113-T7 *ΔentB ΔpabC*	This study
*E. coli* BW-T7/MU	BW25113-T7 containing pMU	This study
*E. coli* BW-T7/MUW	BW25113-T7 containing pMU and pW	This study
*E. coli* BW-T7/MUQ	BW25113-T7 containing pMU and pQ	This study
*E. coli* BW-T7/MUF	BW25113-T7 containing pMU and pF	This study
*E. coli* ΔB/MU	ΔB containing pMU	This study
*E. coli* ΔC/MU	ΔC containing pMU	This study
*E. coli* ΔBC/MU	ΔBC containing pMU	This study
*E. coli* ΔB/MUW	ΔB containing pMU and pW	This study
*E. coli* ΔC/MUW	ΔC containing pMU and pW	This study
*E. coli* ΔBC/MUW	ΔBC containing pMU and pW	This study
*E. coli* ΔB/MUWI	ΔB containing pMU and pWI	This study
Plasmids		
pACYCD-Blank	Cloning vector, CmR, p15a ori	Laboratory stock
pMU	plasmid for biosyntheizing VK_2_ (f1 ori; KanR; *menA; ubiE*; *LacI* gene and T7-LacI promoter)	This study
pQ	pACYCD containing *qorB* genes; T7 promoter	This study
pW	pACYCD containing *wrbA* genes; T7 promoter	This study
pF	pACYCD containing *menF* genes; T7 promoter	This study
pWI	pACYCD containing *wrbA* and *ispB* genes; T7 promoter	This study
pCas	plasmid for CRISPR (temperature sensitive oriR101; KanR; the λ-Red operon under the control of arabinose-inducible promoter; S. pyogenes-derived cas9; sgRNA guided to ori-p15a under the control of lac operator)	Laboratory stock
pTarget-gene	plasmid for CRISPR (p15a ori; CmR; sgRNA guided to targeted gene, such as *entB* or *pabC*)	This study
pACYCD-Donor DNA	pACYCD containing Donor DNA for targeted gene (such as *entB* or *pabC*)	This study

### Growth conditions

All DNA operations were performed using LB medium (10 g/L tryptone, 5 g/L yeast extract, 10 g/L NaCl, pH 7.2). For flask fermentation, the following media were used. Medium M9YE-10G contained 1 g/L NH_4_Cl, 0.5 g/L NaCl, 3 g/L KH_2_PO_4_, 17.1 g/L Na_2_HPO_4_·12H_2_O, 2 mM MgSO_4_, 0.1 mM CaCl_2_, 2 g/L yeast extract, 25 ml/L glycerol and 10 g/L glucose. Medium M9YE-4G contained 1 g/L NH_4_Cl, 0.5 g/L NaCl, 3 g/L KH_2_PO_4_, 17.1 g/L Na_2_HPO_4_·12H_2_O, 2 mM MgSO_4_, 0.1 mM CaCl_2_, 2 g/L yeast extract, 25 ml/L glycerol and 4 g/L glucose. Medium M9-10G contained 10 g/L glucose, which contained 1 g/L NH_4_Cl, 0.5 g/L NaCl, 3 g/L KH_2_PO_4_, 17.1 g/L Na_2_HPO_4_·12H_2_O, 2 mM -MgSO_4_, 0.1 mM CaCl_2_, 25 ml/L glycerol and 10 g/L glucose. For batch fermentation, a modified citric acid medium (CA) was used, which contained 1.86 g/L citric acid, 9 g/L KH_2_SO_4_, 6 g/L (NH_4_)_2_HPO_4_, 0.6 mg/L MgSO_4_, 7.5 mg/L FeSO_4_, 2 g/L yeast extract, 25 mL/L glycerol and 20 g/L glucose, pH 7.0. The antibiotic concentrations and IPTG induction conditions used in this study was showed in Table 2. Considering cell growth and VK_2_ stability, the pH was measured with a glass electrode and controlled at 6.5 ± 0.3 with 4 M NaOH.

**Table 2:** Antibiotic concentrations and IPTG induction conditions

Component	Final concentration	Note
Ampicillin	100 mg/L	Providing selective pressure for Ampicillin resistant plasmid
Chloramphenicol	25 mg/L	Providing selective pressure for Chloramphenicol resistant plasmid
Kanamycin	50 mg/L	Providing selective pressure for Kanamycin resistant plasmid
IPTG	0.1 mM	Inducing expression for flask fermentation
IPTG	0.5 mM	Inducing expression for batch fermentation

All concentrations are expressed as final values in the culture medium. Antibiotics were added at the start of inoculation. IPTG was added to induce expression when cultures reached an optical density of 0.6 (for flask fermentation) or for 8 h after inoculation (for batch fermentation)

### Selection of knockout sites and design of homologous recombination

The functions and detailed information of the *entB* and *pabC* genes were verified in the NCBI and BioCyc databases. The sequence of the *entB* and *pabC* genes in the BW25113-T7 genome was confirmed in NCBI. The N20 site serves as the recognition site for the sgRNA, guiding the Cas9 protein to induce site-specific double-strand breaks (DSBs). The N20 site was identified using the BROAD Institute design tool, which can be accessed at the following website: http://www.broadinstitute.org/rnai/public/analysis-tools/sgrna-design.

### CRISPR plasmid construction and PCR preparation of linear donor DsDNA

Table S1 lists the primer pairs we designed for gene cloning and intermediate plasmid construction. The plasmids pCas (Fig. S1A) and pTarget were prepared in our laboratory. The plasmid pTarget-gene was constructed by reverse transcription polymerase chain reaction and T4 ligation (T4 DNA ligase, NEB, England), with the N20 fragment replaced using specific primers (Fig. S1B). Plasmids pACYCD-gene and pACYCD-Donor-Gene were constructed using the In-Fusion^®^ HD Cloning Kit (Takara, Japan) for the preparation of donor DNA (Figure S2). Donor DNA was cloned from pACYCD-Donor-Gene using Hi-Fi PCR (Phusion^®^ High-Fidelity PCR Master Mix, NEB, England). All plasmids used in this study are listed in Table 1.

### Electroporation, cell recovery and electroplating

Electroporate plasmid or linear DNA into competent cells using a pre-chilled cuvette (0.1 cm) with the Bio-Rad MicroPulser (1.8 kV, time constant > 5.0 ms). Use 25 µg/mL chloramphenicol (Chl) or 50 µg/mL kanamycin (Kan) alone or in combination. Induce λ-Red proteins and the lac vector with 1 mM arabinose and 1 mM IPTG.

Cells cultured at 37 °C (OD_600_ = 0.45–0.55) were energized and electroporated with pCas (100 ng), then recovered in 1 mL SOC medium and incubated at 30 °C for 1 h. The cells were plated on Kanamycin plates and incubated at 30 °C for 18–24 h.

In CRISPR/Cas9-mediated homologous recombination, cells carrying pCas were cultured at 30 °C in medium containing Kan and arabinose to induce competence. After co-electroporation of donor DNA (400 ng) and pTarget-Gene (100 ng), the cells were recovered in SOC medium (1 mL) and incubated at 30 °C for 1 h, then plated on Chl/Kan plates and incubated at 30 °C for 18–24 h.

To eliminate pTarget-gene, cells simultaneously carrying pCas and pTarget were cultured in medium containing Kan and IPTG at 30 °C for 2 h, then plated on Kan plates and incubated at 30 °C for 18–24 h.

To eliminate pCas, cells carrying pCas were cultured in antibiotic-free medium at 37 °C for 12–16 h, and then plated on antibiotic-free plates and incubated at 37 °C for another 12–16 h.

### Confirmation of CRISPR/Cas9-mediated gene knockout in BW25113-T7

Targeted gene modification was rationally performed in *E. coli* BW25113-T7 using CRISPR/Cas9. Two target genes (*entB* and *pabC*) were cloned from the genome of *E. coli* BW25113-T7 to prepare donor DNA. Taking the knockout process of the *entB* gene as an example. Ideally, the *entB* gene knockout profile of BW25113-T7 is shown in Fig. S3. The homologous left arm (HRL) and homologous right arm (HRR) are located near the N20 site. The homologous arms are approximately 400 bp in length, with relatively high recombination efficiency. After CRISPR/Cas9-mediated gene knockout, the target gene will have a 100 bp DNA deletion.

To perform CRISPR/Cas9-mediated homologous recombination in BW25113-T7, we introduced the pCas plasmid, which encodes Cas and λ-Red proteins, into *E. coli* BW25113-T7 via electroporation, and then induced the λ-Red proteins Gam, Bet, and Exo encoded by pCas using arabinose (Ara). After preparing the recipient cells, pTarget-gene (e.g., pTarget-entB) and donor DNA were co-electroporated into the cells (Fig. S4).

To verify the deletion of the target loci, all mutant bacterial strains were subjected to colony PCR. The characteristics of these mutant strains are shown in Table 1.

### Bacterial growth in different culture media

All *E. coli* strains were cultured at 37 °C in 250 mL Erlenmeyer flasks containing M9-10G medium or LB medium. Growth was measured by monitoring optical density at 600 nm (OD_600_) using a spectrophotometer.

The growth rate was fitted to a Sigmoidal-4PL curve. Based on the fitted curve, the OD and the time required to reach the OD were calculated. During the exponential growth phase, a linear fit is performed on ln(OD) versus time. Then the slope of this fit was calculated as growth rate of the strain in the medium (h^− 1^).

### Fermentation cultivation methods

Flask cultivation was carried out in 100 mL Erlenmeyer flasks containing 30 mL of modified minimal medium at 37 °C with shaking at 200 rpm. An inoculum volume of 1% from an overnight culture (12 h) was used. Cultivate in M9YE-10G, M9YE-4G or M9-10G at 37 °C until OD_600_ reaches 0.7. Add IPTG to the induction group to a final concentration of 0.1 mM. Batch fermentation was performed in 5 L fermenter containing 3.5 L CA medium. A 10% (v/v) inoculum from seed cultures was used. Seed cultures were obtained by batch fermentation, which was performed in 5 L fermenter containing 3.5 L CA medium and grown at 37℃ for 12 h, pH 6.9. To obtain seed cultures, a 1% (v/v) inoculum from an overnight culture was used. Glucose as a carbon source was added at initial with a concentration of 20 g/L, while 25 ml/L glycerol was added at initial. Fermentation was operated at 37℃ for 6 h and then IPTG with a final concentration of 0.5 mM was added for induction. After induction, the cultivation temperature was switched to 30℃. The whole fermentation was conducted at 30% dissolved oxygen (automatically adjusted with aeration and agitation rates), and pH 6.5 (automatically adjusted with H_2_SO_4_ and ammonium hydroxide). Solutions of glucose (700 g/L) and glycerol (99.7%) were fed into the fermenter to maintain the glucose concentration between 1 and 10 g/L, and to supplement glycerol with the speed of 0.1–0.2 mL/L/h.

### Analytical procedures

For the crude extract of VK_2_, 2 volumes of 95% ethanol were added to the bacterial culture and then stirred for 30 min. Then the extract was filtered and centrifuged at 6000 rpm for 30 min to collect the supernatant. Vacuum concentrate the supernatant to remove ethanol. Then, add an equal volume of n-butanol to the solution, vigorously shake for extraction, allow phase separation and collect the organic phase to obtain the crude extract.

For the analysis of VK_2_, the crude extract was applied to a C18 reverse-phase column (particle size 5 mm, 4.6 mm 150 mm, Agilent, Santa Clara, CA), and eluted under isocratic conditions with n-butanol at a flow rate of 1 mL/min using an Agilent 1200 HPLC system equipped with a photodiode array detector. The amounts of MKH_2_-8 were quantified by comparing the peak areas with those of concentrations of a standard VK_2_, respectively. VK_2_ (MK-8) was purchased from Aladdin (China) and was dissolved in n-butanol. Six VK_2_ solutions (0, 10, 25, 50, 100, and 200 mg/mL) were prepared and used as standards.

### Data analysis

VK_2_ production data were analyzed using one-way analysis of variance (ANOVA) with GraphPad Prism (version 7.00). Error bars represent the standard error of the mean (SEM). Dunnett’s multiple comparisons test was used to calculated *p*-values were calculated using (*p < 0.05, **p < 0.01, ***p < 0.001). Compare the average value of each column with the average value of the control column. Use strain BW-T7/MU as the control column or parent strain unless otherwise stated.

## Results

### Available metabolic strategy of VK_2_ production in *E. coli* by overexpressing *menA* and *ubiE*

**Fig. 2:**
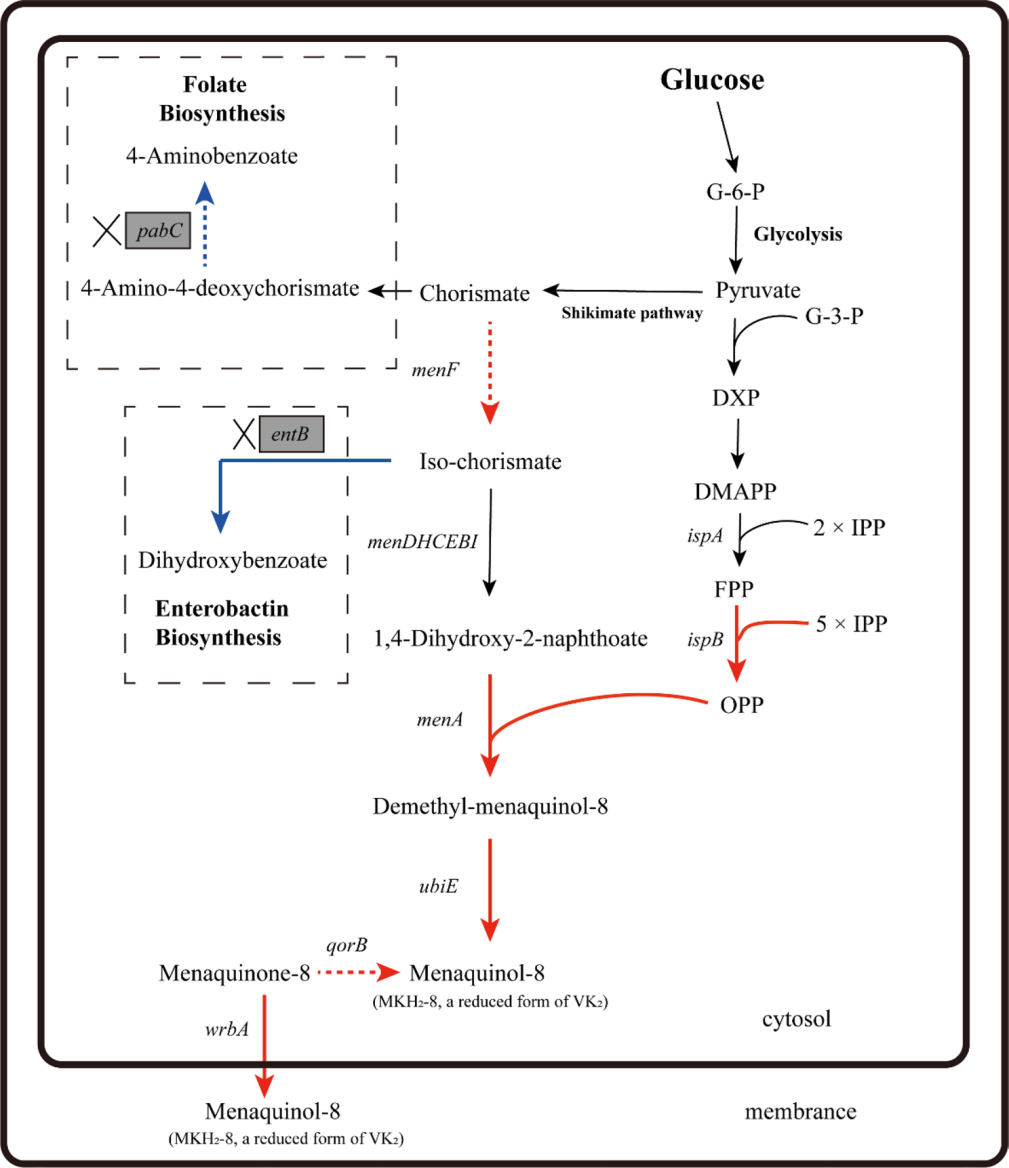
The biosynthetic pathways of VK_2_ in *E. coli* and the strategies for constructing the VK_2_ producing strain. Gene names are placed near the arrow line and italicized. The shaded boxes indicate the genes that were designed to knock out. Red arrows indicate increased flux or activity by directly over-expressing the corresponding genes (full line means expected positive effect on VK_2_ accumulation, while dotted line means no positive effect on VK_2_ accumulation). Blue arrows indicate decreased flux or activity by knocking out the corresponding genes (full line means expected positive effect on VK_2_ accumulation, while dotted line means no positive effect on VK_2_ accumulation)

In previous work, we integrated the T7 expression system into *E. coli* BW25113 and named the resulting strain BW25113-T7. This strain achieved efficient protein expression through the T7 expression system [17, 18]. In this study, the strategy for constructing an engineering strain to efficiently produce VK_2_ involves the overexpression of key genes and rational metabolic engineering. The biosynthetic pathway of VK_2_ in *E. coli* and the strategy for constructing VK_2_-producing strains are shown in Fig. 2. Based on the positions of relevant genes within the biosynthetic pathway, we identified two key genes that are most closely linked to VK_2_ synthesis: *menA* and *ubiE*. The *menA* gene encodes 1,4-dihydroxy-2-naphthoate octaprenyltransferase, an enzyme that catalyzes the transfer of the octaprenyl side chain to DHNA (1,4-dihydroxy-2-naphthoic acid) [20, 21]. The *ubiE* gene encodes a bifunctional 2-octaprenyl-6-methoxy-1,4-benzoquinone methylase and demethylmenaquinone methyltransferase [22]. It encodes a C-methyltransferase capable of catalyzing reactions in the biosynthesis of ubiquinol and menaquinol.

A plasmid to overexpress the key genes *menA* and *ubiE* was constructed and named as pMU. In this plasmid, these two key genes are strictly regulated by the T7-Lac promoter and efficiently expressed. Subsequently, this plasmid pMU was transformed into the BW25113-T7 strain to obtain BW-T7/MU. Then this strain was cultured in M9YE-10G medium. After 24 h and 48 h of induced fermentation, the titers of VK_2_ were determined to be 303 mg/L and 232 mg/L, respectively (Table 3).

**Table 3:** VK_2_ accumulation in Recombinant *E. coli* expressing various related genes

Strain	Plasmid	Expressed genes	VK_2_ accumulation (mg/L)	Fold change	Significance summary	*p* value
24 h fermentation						
BW-T7/MU	pMU	*menA*,* ubiE*	303 ± 1	1.00	/	/
BW-T7/MUQ	pMU + pQ	*menA*,* ubiE*,* qorB*	251 ± 13	0.83	ns	0.792
BW-T7/MUW	pMU + pW	*menA*,* ubiE*,* wrbA*	284 ± 18	0.94	ns	0.999
BW-T7/MUF	pMU + pF	*menA*,* ubiE*,* menF*	158 ± 52	0.52	*	0.018
48 h fermentation						
BW-T7/MU	pMU	*menA*,* ubiE*	232 ± 8	1.00	/	/
BW-T7/MUQ	pMU + pQ	*menA*,* ubiE*,* qorB*	207 ± 19	0.89	ns	0.992
BW-T7/MUW	pMU + pW	*menA*,* ubiE*,* wrbA*	302 ± 55	1.30	ns	0.509
BW-T7/MUF	pMU + pF	*menA*,* ubiE*,* menF*	112 ± 14	0.48	ns	0.060

A 1% (v/v) inoculum from an overnight culture for 12 h was used. IPTG was added when OD_600_ reached 0.7. Samples were taken and measured until 24 h and 48 h, respectively. M9YE-10G medium was used. Glucose was added initially as major carbon source. The results are presented as the mean ± SEM (*n* = 3). BW-T7/MU was set as the parent strain when the fold change was calculated. Dunnett’s multiple comparisons test was used to calculate *p* values (*p < 0.05, **p < 0.01, ***p < 0.001)

### Further overexpression of the *qorB*, *wrbA*, or *menF* genes involved in the menaquinol biosynthesis pathway in *E. coli*

**Fig. 3:**
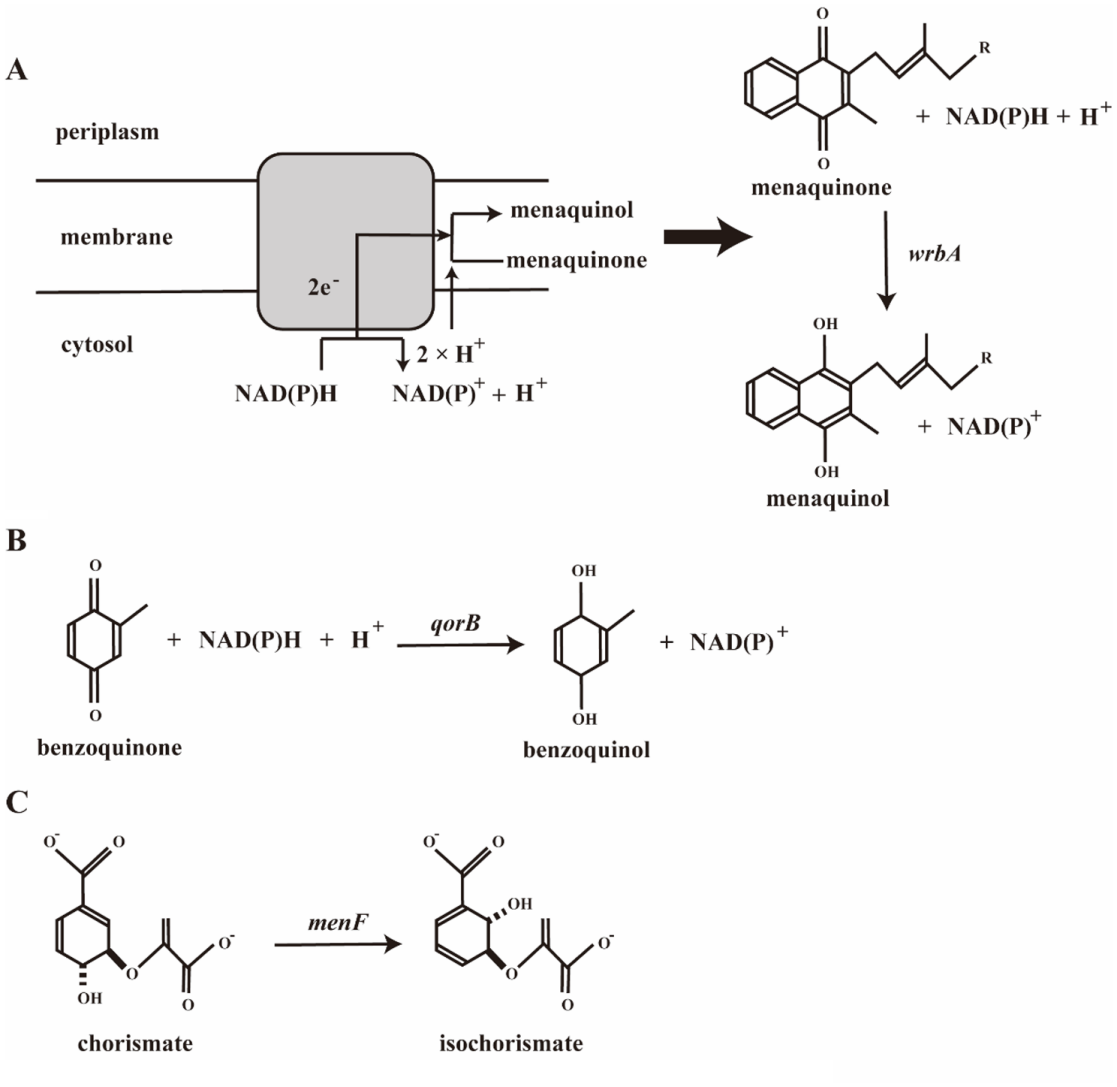
The reactions catalyzed by corresponding genes which managed to overexpress. Gene names are placed near the arrow line and italicized. The reaction directions shown in accordance with the physiological direction of the reaction. (**A**) The *wrbA* gene encodes the enzyme that catalyzes redox reaction of quinones in membrane. (**B**) The *qorB* gene encodes the enzyme that catalyzes redox reaction of quinones in cytosol. (**C**) The *menF* gene encodes the enzyme that catalyzes the conversion of chorismate to isochorismate

To increase the levels of isoprenoid acids used for MK synthesis and MKH_2_, we further overexpressed the *wrbA*, *qorB* and *menF* genes individually. The reactions catalyzed by these genes are shown in Fig. 3.

In our study, we constructed three plasmids (pW, pQ and pF) to overexpress these three genes individually (Table 1). Then these three plasmids are individually introduced into *E.coli* strain BW-T7/MU to obtain BW-T7/MUW, BW-T7/MUQ and BW-T7/MUF. These obtained strains were cultured in a modified medium M9YE-10G for VK_2_ accumulation analysis. VK_2_ concentrations were measured after 24 h and 48 h of fermentation. The strain BW-T7/MU containing only pMU was used as the control.

**Fig. 4:**
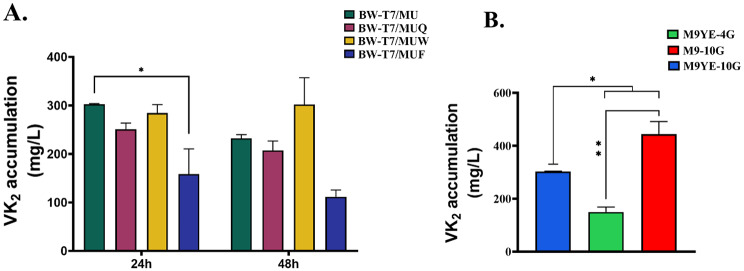
**(A)** VK_2_ accumulation in recombinant *E. coli* expressing various related genes in flask fermentation. Strains: BW-T7/MU (BW25113-T7 harboring pMU), BW-T7/MUQ (BW25113-T7 harboring pMU and pQ), BW-T7/MUW (BW25113-T7 harboring pMU and pW), BW-T7/MUF (BW25113-T7 harboring pMU and pF). Cultivation was performed in 300 mL Erlenmeyer flask supplied with 30 mL M9-10G medium for 24 h and 48 h, respectively. Then the final Vk_2_ accumulation result was measured. Results are the average of three individual experiments. Error bar indicated standard error (SEM). **(B)** The production of VK_2_ by recombinant *E. coli* BW25113-T7 harboring the pMU plasmid (BW-T7/MU) was assessed following a 24 h incubation in various media (M9YE-10G, M9YE-4G and M9-10G). Results are the average of three individual experiments. Error bar indicated standard error (SEM). Dunnett’s multiple comparisons test was used to calculate *p* values (*p < 0.05, **p < 0.01, ***p < 0.001)

Based on our results (as shown in Table 3; Fig. 4A), all strains carrying additional plasmids (namely BW-T7/MUF, BW-T7/MUQ, and BW-T7/MUW) exhibited a reduction in VK_2_ accumulation after 24 h of fermentation. We found that strain BW-T7/MUW showed a favorable increasing trend in VK_2_ titer after 48 h of fermentation, which corresponds to a 1.30-fold increase over the parental strain (BW-T7/MU). However, the VK_2_ titer of the other strains showed a decreasing trend at 48 h.

### Optimization of medium for flask fermentation

While optimizing the metabolic pathway of the strain, we cultured the chassis strain BW-T7/MU in three different media formulations (M9YE-10G, M9YE-4G and M9-10G). The research results (as shown in Table 4; Fig. 4B) revealed that after adjusting the medium composition, the accumulation of VK_2_ by the strain in M9-10G medium was significantly increased compared to the previous M9YE-10G medium, with a VK_2_ accumulation of 443 mg/L. However, reducing the glucose content significantly decreased the VK_2_ titer of the strain. Therefore, M9-10G medium was used for fermentation culture of the strain in subsequent flask cultivation.

**Table 4:** VK_2_ accumulation in recombinant *E. coli* in different medium

Strain	Expressed genes	Medium	VK_2_ accumulation (mg/L)	Relative Change	Significance summary	*p* value
BW-T7/MU	*menA*,* ubiE*	M9YE-10G	303 ± 1	1.00	/	/
BW-T7/MU	*menA*,* ubiE*	M9YE-4G	150 ± 18	0.49	*	0.020
BW-T7/MU	*menA*,* ubiE*	M9-10G	443 ± 48	1.46	*	0.028

A 1% (v/v) inoculum from an overnight culture for 12 h was used. IPTG was added when OD_600_ reached 0.7. Samples were taken and measured until 24 h. Glucose was added initially as major carbon source. The results are presented as the mean ± SEM (*n* = 3). Dunnett’s multiple comparisons test was used to calculate *p* values (*p < 0.05, **p < 0.01, ***p < 0.001)

### Enhancing VK_2_ production through modification of the metabolic pathway

To further improve the BW25113-T7 strain, we carried out the following targeted genetic modifications.

**Fig. 5:**
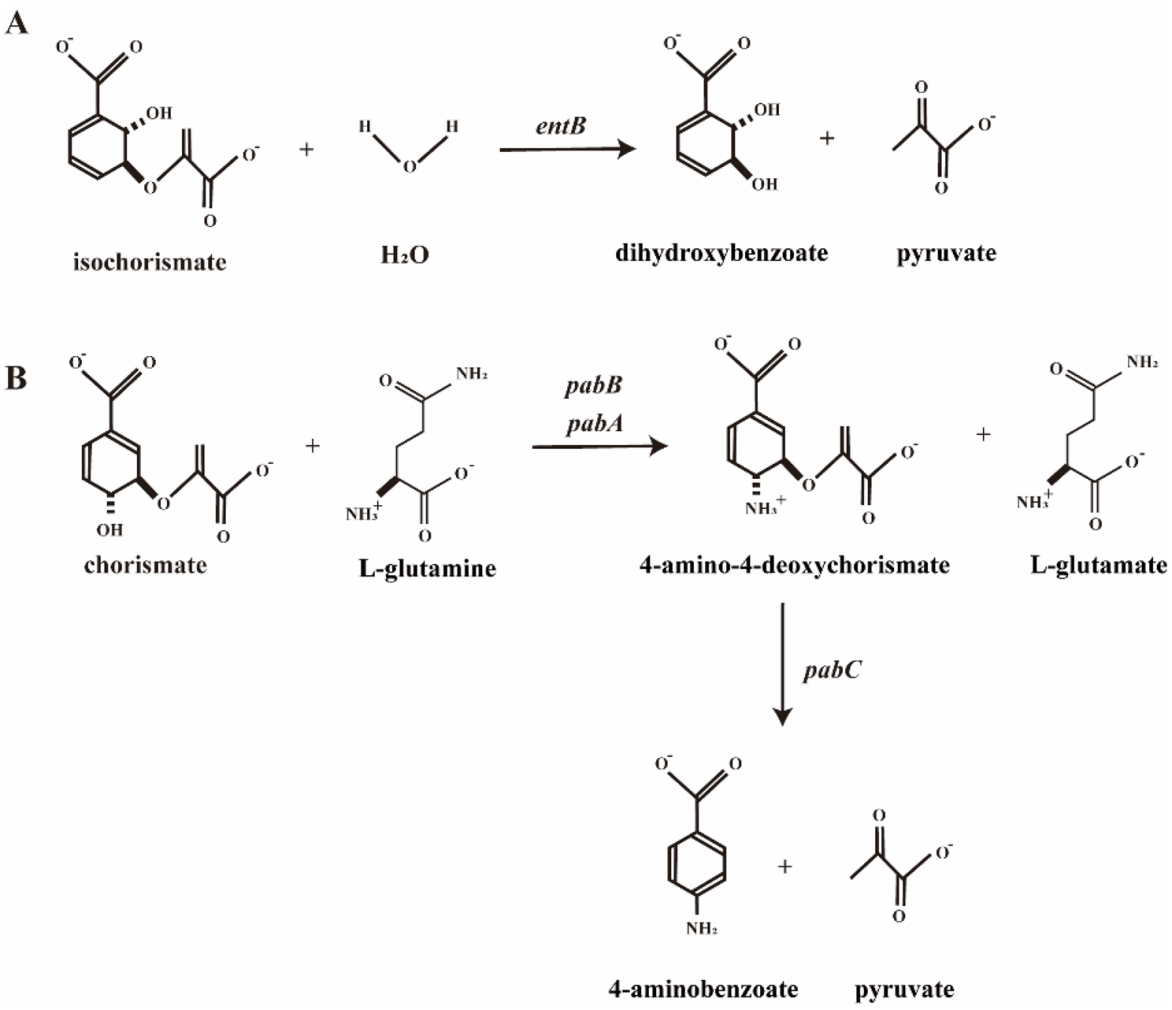
The reactions catalyzed by corresponding enzymes. Gene names are placed near the arrow line and italicized. The reaction directions shown in accordance with the physiological direction of the reaction. (**A**) The synthesis of 2,3-dihydroxy-2,3-dihydrobenzoate catalyzed by EntB. (**B**) The conversion of 4-amino-4-deoxychorismate to 4-aminobenzoate catalyzed by PabC

In the MK pathway of VK_2_ synthesis, the biosynthesis of tetrahydrofolate and enterobactin competes with the substrates for VK_2_ synthesis, as they share the same precursors (chorismate and isochorismate). Therefore, we designed to knock out these genes to direct more substrates toward the menaquinol synthesis pathway. As shown in Fig. 5A, *entB* encodes an isochorismatase that catalyzes the synthesis of 2,3-dihydroxybenzoic acid and is also involved in the biosynthesis of enterobactin [23]. The *pabC* gene encodes aminodeoxychorismatelyase. The formation of 4-amino-4-deoxychorismate from chorismate is catalyzed by *pabB* and *pabA* (both these two genes are essential for growth of *E. coli* in M9 minimal medium), while *pabC* catalyzes the conversion of 4-amino-4-deoxychorismate to 4-aminobenzoate (Fig. 5B), which participates in the synthesis of folate [24–26].

**Fig. 6:**
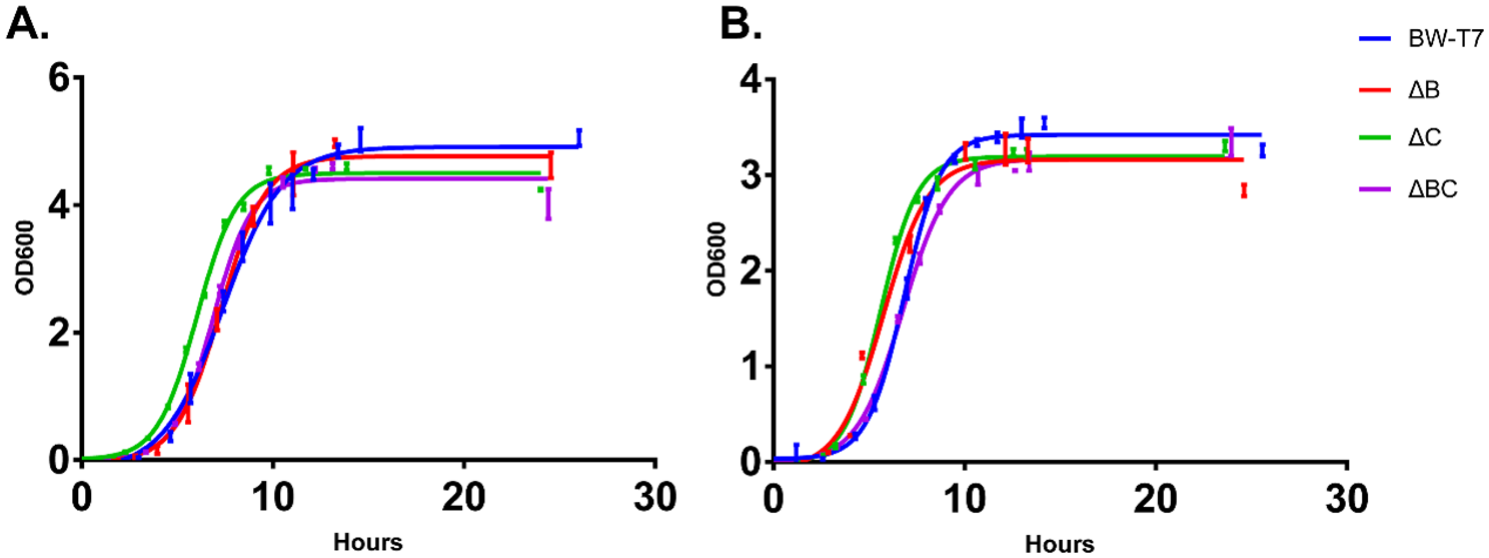
Growth characteristic of mutant *E. coli* strains in different medium. Strains: BW-T7 (BW25113-T7), ΔB (BW25113-T7 Δ*entB*), ΔC (BW25113-T7 Δ*pabC*), ΔBC (BW25113-T7 Δ*entB* Δ*pabC*). Cells were cultured in LB (**A**) or M9-10G (**B**) medium. Data are means of three replicates. Error bar indicated standard deviation

In our study, we performed individual and combined knockouts of the *entB* and *pabC* genes in *E. coli* strain BW25113-T7. Growth of these mutant *E. coli* strains in different medium was examined to assess whether CRISPR/Cas9-mediated gene knock-out affected the metabolic characteristics of the bacteria (Fig. 6). There were no differences in growth rate among these strains (BW-T7, ΔB, ΔC and ΔBC) in LB and M9-10G medium (Table S2).

**Fig. 7:**
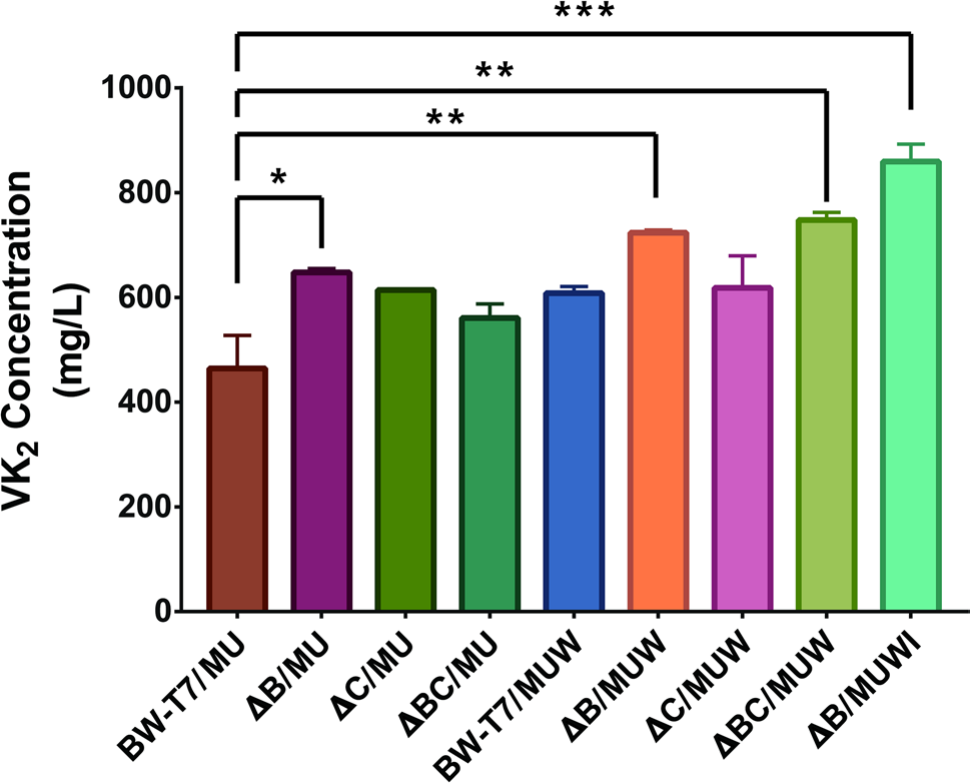
VK_2_ production in different *E. coli* mutant strains. Strains: BW-T7/MU (BW25113-T7 harboring pMU), ΔB/MU (ΔB harboring pMU), ΔC/MU (ΔC harboring pMU), ΔBC/MU (ΔBC harboring pMU), BW-T7/MUW (BW25113-T7 harboring pMU and pW), ΔB/MUW (ΔB harboring pMU and pW), ΔC/MUW (ΔC harboring pMU and pW), ΔBC/MUW (ΔBC harboring pMU and pW) ΔB/MUWI (ΔB harboring pMU and pWI). All strains were incubated in M9-10G medium for 48 h. Results are the average of three individual experiments. Error bar indicated standard error (SEM). Dunnett’s multiple comparisons test was used to calculate *p* values (*p < 0.05, **p < 0.01, ***p < 0.001)

We subsequently introduced the plasmid pMU or dual plasmids (pMU and pW) into the mutant strains after gene knockout. After 48 h of fermentation in M9-10G medium, the fermentation broth was extracted and the accumulation of VK_2_ was detected. As shown in Table 5; Fig. 7, the VK_2_ accumulation levels of ΔB/MU and ΔB/MUW were 648 mg/L and 724 mg/L, respectively. The VK_2_ accumulation level of the mutant strain ΔBC/MUW was significantly higher than that of the control strain BW-T7/MU (*p* < 0.01), reaching a concentration of 748 mg/L. However, no significant difference was found between ΔB/MUW and ΔBC/MUW (*p* = 0.9994).

**Table 5:** VK_2_ accumulation of different mutant strains

Strain	Relevant characteristic(s)	Expressed genes	VK_2_ accumulation (mg/L)	Fold change	Significance Summary	*p* value
BW-T7/MU	BW25113-T7	*menA*,* ubiE*	464 ± 63	1.00		
ΔB/MU	BW25113-T7 *ΔentB*	*menA*,* ubiE*	648 ± 8	1.38	*	0.045
ΔC/MU	BW25113-T7 *ΔpabC*	*menA*,* ubiE*	614 ± 2	1.31	ns	0.128
ΔBC/MU	BW25113-T7 *ΔentB ΔpabC*	*menA*,* ubiE*	561 ± 27	1.20	ns	0.504
BW-T7/MUW	BW25113-T7	*menA*,* ubiE*,* wrbA*	608 ± 13	1.30	ns	0.152
ΔB/MUW	BW25113-T7 *ΔentB*	*menA*,* ubiE*,* wrbA*	724 ± 5	1.55	**	0.003
ΔC/MUW	BW25113-T7 *ΔpabC*	*menA*,* ubiE*,* wrbA*	618 ± 61	1.32	ns	0.113
ΔBC/MUW	BW25113-T7 *ΔentB ΔpabC*	*menA*,* ubiE*,* wrbA*	748 ± 15	1.60	**	0.001
ΔB/MUWI	BW25113-T7 *ΔentB*	*menA*,* ubiE*,* wrbA*,* ispB*	859 ± 15	1.85	***	< 0.001

A 1% (v/v) inoculum from an overnight culture for 12 h was used. IPTG was added when OD_600_ reached 0.7. Samples were taken and measured until 48 h. 10 g/L glucose was added initially as major carbon source. The results are presented as the mean ± SEM (*n* = 3). BW-T7/MU was set as the parent strain when the fold change was calculated. Dunnett’s multiple comparisons test was used to calculate *p* values (*p < 0.05, **p < 0.01, ***p < 0.001)

### Overexpression of *ispB* gene and batch fermentation

**Fig. 8:**
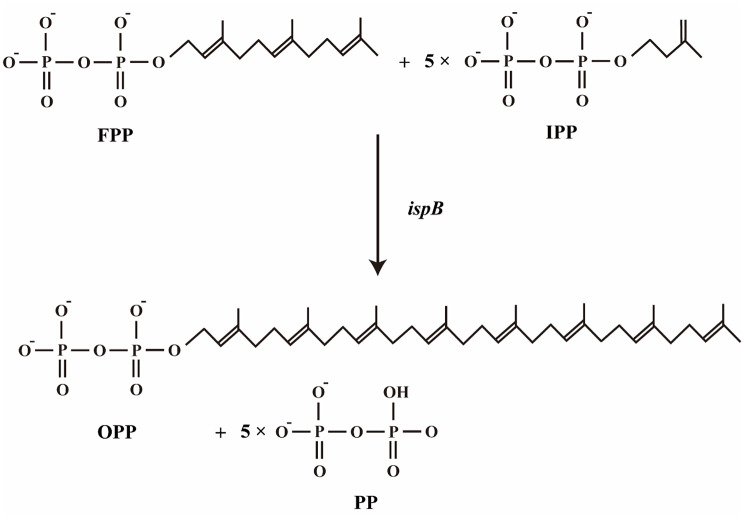
The reactions catalyzed by IspB. The reaction directions shown in accordance with the physiological direction of the reaction. FPP: farnesyl diphosphate. IPP: isopentenyl diphosphate. OPP: octaprenyl diphosphate. PP: diphosphate

The synthesis of MKH_2_-8 can be divided into two parts: the synthesis of the naphthoquinone ring and the isoprenoid side chain (C40). To further enhance VK_2_ production, we overexpressed *ispB* gene in the recombinant strain ΔB/MUW. This gene encodes an enzyme which could catalyze the condensation reactions resulting in the formation of all-trans-octaprenyl diphosphate, the isoprenoid side chain of ubiquinol-8 and MKH_2_-8 (Fig. 8). The enzyme adds five isopentenyl diphosphate molecules sequentially to farnesyl diphosphate with trans stereochemistry [27, 28]. The reaction provides the isoprenoid side chain for the synthesis of MKH_2_-8. Therefore, we believe that overexpression of this gene can increase the synthetic flux of MKH_2_-8. In this study, we constructed a new plasmid pWI, which overexpressed *wrbA* and *ispB* synergistically. Subsequently, pMU and pWI were both introduced to *E. coli* ΔB to obtain ΔB/MUWI. After 48 h of fermentation in M9‑10 G medium, strain ΔB/MUWI reached a VK_2_ titer of 859 mg/L, which is a 1.85‑fold increase over the parent strain BW‑T7/MU.

**Fig. 9:**
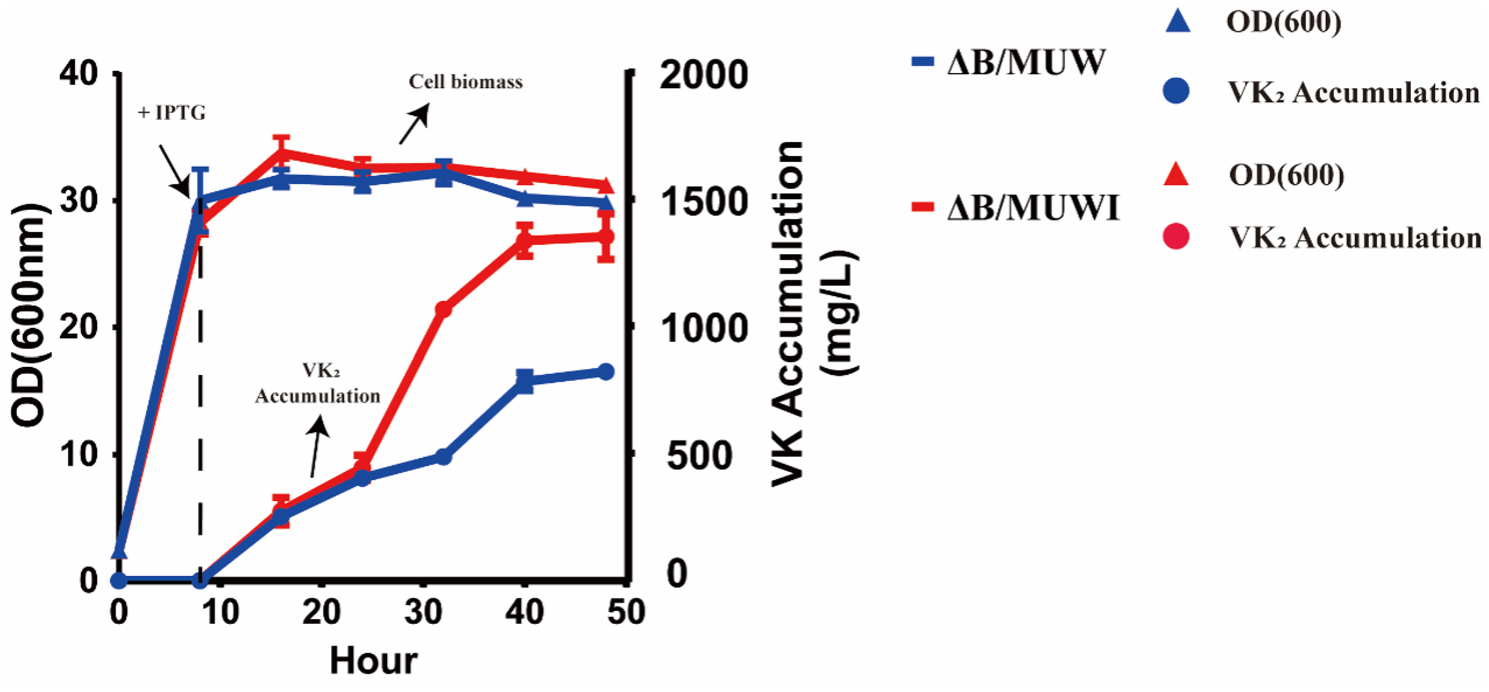
Batch fermentation of VK_2_ in *E. coli* ΔB/MUW and ΔB/MUWI) in a 5 L fermenter. Strains: ΔB/MUW (ΔB harboring pMU and pW), ΔB/MUWI (ΔB harboring pMU and pWI). A 10% (v/v) inoculum from seed cultures was used. 20 g/L glucose and 25 ml/L glycerol were added initially. 0.5 mM IPTG was added as indicated. During the fermentation, the pH was controlled optimally at 6.5. Results are the average of three individual experiments. Error bar indicated standard deviation

Then these two strains (ΔB/MUW and ΔB/MUWI) were fermented in 5 L fermenter and glucose was added with an initial concentration of 20 g/L. After 48 h cultivation, a high titer of VK_2_ (1360 mg/L) was achieved by ΔB/MUWI (Table 6; Fig. 9).

**Table 6:** Batch fermentation of VK_2_ in *E. coli* ΔB/MUW and ΔB/MUWI in a 5 L fermenter

Strain	Relevant characteristic(s)	Expressed genes	Cell biomass (OD_600_)	VK_2_ accumulation (mg/L)
ΔB/MUW	BW25113-T7 *ΔentB*	*menA*,* ubiE*,* wrbA*	29.81 ± 0.25	825 ± 27
ΔB/MUWI	BW25113-T7 *ΔentB*	*menA*,* ubiE*,* wrbA*,* ispB*	31.22 ± 0.42	1360 ± 89

A 10% (v/v) inoculum from seed cultures was used. 20 g/L glucose and 25 ml/L glycerol were added initially. 0.5 mM IPTG was added as indicated. During the fermentation, the pH was controlled optimally at 6.5. The results are presented as the mean ± SEM (*n* = 3)

## Discussion

**Fig. 10:**
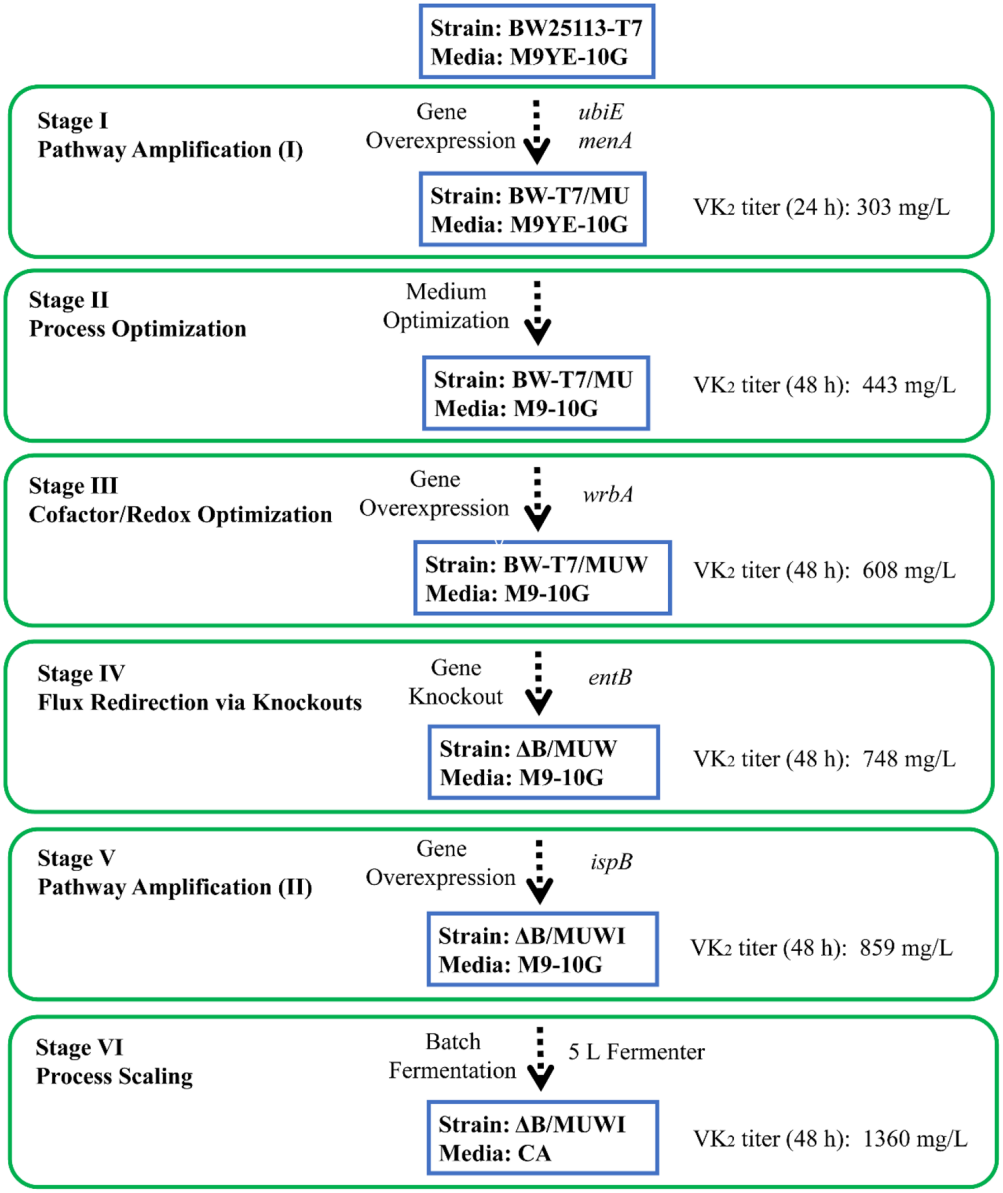
Stepwise engineering strategy for VK_2_ accumulation in recombinant *E. coli* strain. Gene names are placed near the arrow line and italicized

Metabolic engineering provides a powerful tool for regulating metabolic pathways to accumulate desired compounds. Currently, many bio-based products are produced in *E. coli* through metabolic engineering or synthetic biology. In the present study, we developed a metabolic strategy for the aerobic production of VK_2_ using *E. coli* as the host organism and glucose as the carbon source. Leveraging established metabolic regulation information and CRISPR-Cas9 mediated gene knockout, we constructed a well-defined genetically engineered *E. coli* strain. Menaquinol-8 (MKH_2_-8), also known as active form and of VK_2_ [29], is capable of donating electrons and protecting cells from oxidative stress-induced damage (Fig. 1). MKH_2_ is the ultimate target product of our metabolic strategy. The stepwise engineering strategy for VK_2_ accumulation in this study was shown in Fig. 10. In this study, we identified effective genes (*ubiE*, *menA*, *wrbA* and *ispB*) and employed CRISPR/Cas9 to knock out competing‑pathway gene (*entB*). Using this combined strategy, we achieved a VK_2_ accumulation of 1360 mg/L in a 5 L fermenter. Significantly, the engineered strain developed in this study can be further optimized, as all modifications were clearly defined. By replacing or introducing additional key enzymes, the system can be re‑programmed through targeted metabolic‑pathway engineering. This modularity enables the biosynthesis of other quinones (e.g., ubiquinone, plastoquinone) or isoprenoid compounds (e.g., carotenoids, terpenes).

We first constructed the VK_2_-producing *E. coli* base strain by amplifying two key genes. As mentioned earlier, the *menA* gene encodes a key prenyltransferase that catalyzes the formation of demethylmenaquinol‑8 (DMKH_2_‑8) [20, 21]. This rate-limiting step in VK_2_ biosynthesis serves as the metabolic junction between the shikimate pathway (DHNA production) and the methylerythritol phosphate (MEP) pathway (isopentenyl diphosphate synthesis). By transferring the isoprenoid group to DHNA, the reaction establishes the fundamental scaffold of DMKH_2_-8, a direct precursor to bioactive menaquinones. Another key gene, *ubiE*, encodes the enzyme that methylates DMKH_2_‑8 to MKH_2_‑8, the last step in menaquinol biosynthesis [22]. It directly determines the production of menaquinol and plays a crucial concluding role in the entire biosynthetic process. We overexpressed *menA* and *ubiE* genes using T7 system in *E. coli* BW25113-T7 and achieved a VK₂ concentration of 303 mg/L after 24 h of flask fermentation (Table 3).

Subsequently, we identified three candidate genes—*wrbA*, *qorB*, and *menF*—that may have a positive effect on MKH_2_-8 accumulation through metabolic network analysis. The flavin-dependent quinone oxidoreductase WrbA [30] plays a dual role in MK-8 biosynthesis. First, it maintains intracellular redox homeostasis by reducing polycyclic quinone intermediates, including oxidized forms of DHNA [31]. Second, WrbA mitigates oxidative stress by diminishing reactive oxygen species (ROS) accumulation through its participation in quinone reduction reactions, thereby protecting cells from oxidative damage [32]. WrbA could efficiently transfer electrons from NAD(P)H to quinone substrates. In the biosynthetic pathway of VK_2_ in *E. coli*, MK‑8 can be converted to its reduced form MKH_2_‑8 and be further utilized or stored. We believe that overexpression of *wrbA* could accelerate the reduction step from MK‑8 to MKH_2_‑8, thereby increasing the pool of reduced quinone and directly boosting the final VK_2_ accumulation. In addition, WrbA enables the accumulation of intracellular substances to be concentrated on the membrane through quinone redox reactions (Fig. 3A). The VK_2_ produced by *E. coli* mainly binds to its cell membrane [33]. Therefore, we believe that overexpressing the *wrbA* gene can facilitate the enrichment of VK_2_ in the membrane, reduce its accumulation in the cytosol and enhance the accumulation efficiency of VK_2_. In this study, we found that the recombinant strain with overexpression of *wrbA* (strain BW-T7/MUW) exhibited a 1.30‑fold increase in VK_2_ accumulation compared with the parental strain BW‑T7/MU. This result indicates that overexpression of the *wrbA* gene in *E. coli* may lead to positive results in improving menaquinol production.

The NAD(P)H-quinone oxidoreductase (encoded by *qorB* gene) catalyzes the reduction of quinones (Fig. 3B) [34, 35]. Studies have shown that *E.coli* strains overexpressing *qorB* exhibit growth defects and a significant decrease in several enzymes involved in carbon metabolism [35]. Additionally, MKH_2_-8 is not a strict substrate for this oxidoreductase. The reduction of VK_2_ accumulation in *E. coli* BW-T7/MUQ might be related to these reasons.

When synthesizing MK from glucose in *E. coli*, chorismate serves as a critical branching point in the biosynthetic pathway. A portion of the chorismic acid is regulated through enzyme catalysis encoded by the *menFDCEBA* gene cluster to produce key precursors for MK synthesis. Another portion is diverted through competing pathways to generate other metabolites. Among them, isochorismate is an important precursor in the MK pathway synthesis. Isochorismate synthase encoded by *menF* catalyzes the isomerization of chorismate to isochorismate (Fig. 3C), which is the first key step in menaquinol biosynthesis via the MK pathway [36]. However, we observed that overexpression of *menF* significantly reduced the accumulation of VK_2_. This result is inconsistent with the previous findings by Kong and Lee [15]. In their study, the overexpression of the seven head structural genes (i.e., *men* cluster genes) in MK-8 all had a positive effect on the accumulation of MK-8. As we apply the T7 expression system to overexpress the most related genes *menA* and *ubiE*, the translational machinery of *E. coli* was rapidly saturated [37]. This saturation of the translational machinery might have inhibited the expression of *menF*, competed with the metabolic flux and thus led to a reduction in MKH_2_-8 synthesis. Among these three related genes, only the overexpression of *wrbA* exhibited a favorable increasing trend in VK_2_ accumulation after 48 h of cultivation in our strategy (Table 3; Fig. 4A).

We hypothesized that culture medium limitations might restrict gene expression. Subsequently, we optimized the medium to determine whether its composition affected VK_2_ accumulation in *E. coli* (Table 4; Fig. 4B). To investigate the effect of varying glucose concentrations in the medium on VK_2_ accumulation, we adjusted the medium by reducing glucose from 10 g/L to 4 g/L. The results show that lowering the glucose content significantly reduces the accumulation of VK_2_. This result also provides a reference for determining the glucose concentration when using CA medium in batch fermentation. Yeast extract is a complex mixture, which contain amino acids, vitamins, nucleotides and trace metal ions. Some metabolites present in yeast extract may act as negative effectors of the pathway by inhibiting key enzymes such as UbiE or MenA. In addition, high concentrations of nutrients might inhibit the accumulation of secondary metabolites in special conditions. Previous study has shown that during the fermentation of *B. subtilis*, excessive yeast extract actually suppresses VK_2_ production [38]. Thus, we hypothesize that the abundant nutrients in yeast extract promote rapid cell growth, which may cause the cells to divert more carbon flux toward biomass synthesis rather than the accumulation of the metabolite VK_2_. Besides, researchers have proved that the ratio of carbon to nitrogen (C/N ratio) is a significant factor affecting VK_2_ production in *B. subtilis* [39]. In our study, the removal of yeast extract reduces the nitrogen source content in the medium, thereby increasing C/N ratio. As a result, the cell growth rate decreases, leading to a reduced demand for ATP. We hypothesize that this metabolic shift likely redirects acetyl-CoA away from the TCA cycle towards the MVA pathway. Consequently, more IPP and DMAPP (dimethylallyl diphosphate) become available precursors for VK_2_ synthesis. Following this, we modified the medium composition by removing yeast extract. While maintaining constant glucose levels, this adjustment significantly increased VK_2_ accumulation, establishing an optimized foundation for subsequent fermentation.

Further improvement of the VK_2_-accumulation strain was achieved to modify the metabolic pathway rationally by using the metabolic and regulatory information available in the literature. As mentioned before, chorismate and isochorismate are key intermediates in menaquinol biosynthesis. Therefore, it is important to increase the substrates for menaquinol synthesis by knocking out genes in the competing pathways. There are so diverse metabolites that use chorismate and isochorismate as key precursors for biosynthesis. In this study, we chose to target tetrahydrofolate and enterobactin for repression of their biosynthesis. The biosynthetic pathways for tetrahydrofolate and enterobactin have been extensively studied, and their key enzymes and genes are relatively well defined. Based on KEGG and BioCyc database, the impact of knocking out critical genes on growth and metabolism of the host strain is relatively controllable. The isochorismate enzyme EntB catalyzes the formation of 2,3-dihydroxybenzoic acid (Fig. 5A) and plays a key role in the enterobactin biosynthesis pathway. It also functions as an aryl carrier protein (ArCP) during enterobactin assembly [23]. Furthermore, *entB* acts synergistically with *entC* to enhance enterobactin synthesis efficiency [40]. Knockout of the e*ntB* gene reduces enterobactin biosynthesis, thereby redirecting metabolic flux toward menaquinol synthesis and consequently enhancing VK₂ production. Our results revealed that the mutant strain with the *entB* gene knocked out alone has a significant increase in the accumulation of VK_2_ compared to the original strain, suggesting that inhibition of enterobactin metabolism has a positive effect on menaquinol biosynthesis.

In *E. coli*, the *pabC* gene encodes the amino deoxy branch acid lyase in the para-aminobenzoic acid synthesis pathway (Fig. 5B), participating in the synthesis of para-aminobenzoic acid (PABA). The synthesis of PABA begins with chorismate and glutamine as the initial substrates. Under the action of the PabA and PabB proteins, 4-amino-4-deoxychorismate (ADC) is first produced [41], which is then converted into PABA and pyruvate by the action of aminodeoxychorismate lyase encoded by the *pabC* gene [24–26]. PABA is an important precursor for folate synthesis, and folate is ultimately converted into its biologically active form through the action of a series of enzymes, participating in various essential metabolic processes. Thus, we hypothesized that the knockout of the *pabC* gene could reduce the synthesis of para-aminobenzoic acid, which in turn may direct metabolic fluxes more towards the synthesis of MKH_2_-8. However, our findings indicate that *pabC* gene knockout alone did not significantly enhance VK_2_ accumulation in the mutant strain. In this case, we speculated that *pabC* knockout alone might have limited impact on regulating the metabolic flux of chorismate (direct precursor of the MKH_2_‑8 biosynthetic route). Besides, we hypothesize that alternative chorismate-consuming pathways may exist, or that *pabC* knockout may induce cellular compensatory responses. Such responses could activate regulatory mechanisms that indirectly impede the MKH_2_-8 biosynthetic pathway, thereby partially offsetting the anticipated promotive effect of increased substrate flux. Consequently, the single knockout of *pabC* does not yield a noticeable positive effect on VK_2_ accumulation.

After attempting the individual knockout of the *pabC* gene and the *entB* gene, we then performed a combined knockout of the two genes. According to the research results, when the two genes were simultaneously knocked out, the mutant strain ΔBC/MUW produced an VK_2_ titer as high as 748 mg/L after 48 h of cultivation in a flask. However, to avoid substantial metabolic burdens from multiple gene knockouts, we retained only those significantly increasing target product titer, thereby maintaining engineered strain robustness. Our results demonstrate that *entB* knockdown enhanced VK_2_ production significantly more than *pabC* knockdown. Furthermore, the VK_2_ accumulation of the *entB* single-knockout strain ΔB/MUW (724 mg/L) showed no statistically significant difference from that of the double knockout strain ΔBC/MUW (748 mg/L). Therefore, we ultimately selected the recombinant strain ΔB/MUW as our dominant strain for the further optimization. Strain ΔB/MUW exhibited a 1.55‑fold increase in VK_2_ accumulation compared with the parental strain BW‑T7/MU.

The synthesis of MKH_2_-8 included two parts: the synthesis of the naphthoquinone ring and the isoprenoid side chain. Previous work in this study has focused on enhancing the synthetic pathway of the naphthoquinone ring. To further enhance the synthesis flux of MKH_2_-8, we attempted to strengthen the synthesis of the isoprenoid side chain. The targeted gene *ispB*, which encoded octaprenyl diphosphate (OPP) synthase, was overexpressed. This enzyme catalyzes the condensation reactions resulting in the formation of OPP, the isoprenoid side chain of ubiquinol-8 and MKH_2_-8. We hypothesize that the overexpression of *ispB* gene will cause a positive effect on MKH_2_-8 biosynthesis and lead to an increased VK_2_ accumulation in recombinant strain ΔB/MUWI. After 48 h of flask fermentation, strain ΔB/MUWI achieved a high VK_2_ titer of 859 mg/L, which corresponds to a 1.85-fold increase over the parental strain (BW-T7/MU).

To accumulate VK_2_ in these recombinant *E. coli* strains more efficiently, we optimized the cultivation condition and culture medium in batch fermentation (optimization process was omitted). After optimization, the VK_2_ accumulation was further enhanced in our modified batch fermentation (see “Materials and Methods” section). Cultivation of ΔB/MUW in modified batch fermentation (825 mg/L) accumulated more VK_2_ than that in flask fermentation (724 mg/L). VK_2_ accumulation in the strain ΔB/MUWI has achieved 1360 mg/L after 48 h cultivation in our modified batch fermentation, which showed the highest VK_2_ titer (Table 6; Fig. 9). These results revealed that enhancing the synthesis of the isoprenoid side chain can effectively increase the accumulation of VK_2_. In addition, optimizing the MEP pathway or the MVA pathway to increase the amounts of DMAPP and IPP, which are important precursors of VK_2_, is also a crucial strategy for the biosynthesis of VK_2_. Previous research has demonstrated that modifying the IPP metabolic pathway could successfully enhance the biosynthesis efficiency of terpenoids in *Bacillus subtilis* and thus improve the accumulation of MK-7 [13]. As a key precursor in the isoprenoid metabolic pathway, optimizing the IPP metabolic pathway can effectively direct the carbon flux towards terpenoid synthesis. This achievement provides valuable insights for our subsequent research on MKH_2_-8 synthesis, suggesting that we can optimize the isoprenoid pathway through similar strategies to improve the synthesis efficiency of MKH_2_-8. It also offers a valuable reference for the biosynthesis of terpenoids.

As indicated in Fig. 10, the VK_2_ titer of best strain we constructed reached 1360 mg/L. However, there remains considerable potential for further increasing the VK_2_ titer in the fermenter. In our laboratory, previous experiments in batch fermentation mainly employed the CA medium, which has become a relatively mature system. Consequently, the present VK_2_ experiments in batch fermentation were conducted using the CA medium as a baseline, with minor adjustments to the formulation and process. However, CA medium may not be the most suitable for VK_2_ accumulation in *E. coli*. Moreover, there remains considerable room for improvement in dissolved oxygen, temperature, feed rate, and other parameters. Besides, we believe that there remains potential for further enhancing VK_2_ titer, encompassing strategies such as “rational design of key enzymes” and “systematic modification of metabolic pathways”. As our work continues, we are actively optimizing the biosynthesis of MKH_2_-8 to create highly competitive engineered strains. Additionally, we are confident that further optimizing the fermentation process will elevate VK_2_ accumulation to even greater heights.

## Conclusion

In summary, rational metabolic engineering based on known metabolic and regulatory information, as well as key gene expression, can lead to the development of *E. coli* strains for the efficient production of VK_2_. A high titer of 1360 mg VK_2_ per liter could be achieved in batch fermentation using metabolically engineered strains. Further optimization of the fermentation process was able to increase the titer of VK_2_ to even higher levels. Besides, the engineered framework constitutes a modular chassis. By replacing or introducing other target key enzymes in this system and performing targeted metabolic-pathway engineering, this modularity can be readily re-programmed for the biosynthesis of other quinones or isoprenoid compounds. This scalable modular chassis highlights the translational relevance of our approach for the industrial biomanufacturing of high‑value metabolites.

## Supplementary Information

Below is the link to the electronic supplementary material.

Supplementary Material 1

## Data Availability

Most of the data generated or analyzed in this study are presented in this published article. Additional data not included here are accessible upon reasonable request to the corresponding author.

## Declarations

### Generative AI

No AI tools were used in this article.
