# Supplementary material for: Metabolic engineering of Escherichia coli BW25113 for the production of Vitamin K2 based on CRISPR/Cas9 mediated gene knockout and metabolic pathway modification
Source: J Biol Eng. 2026 Jan 8;20:29. doi: 10.1186/s13036-025-00614-9 (PMC12879462; doi:10.1186/s13036-025-00614-9)
Supplement: Supplementary file 1 — Supplementary Material 1 [file 13036_2025_614_MOESM1_ESM.docx]

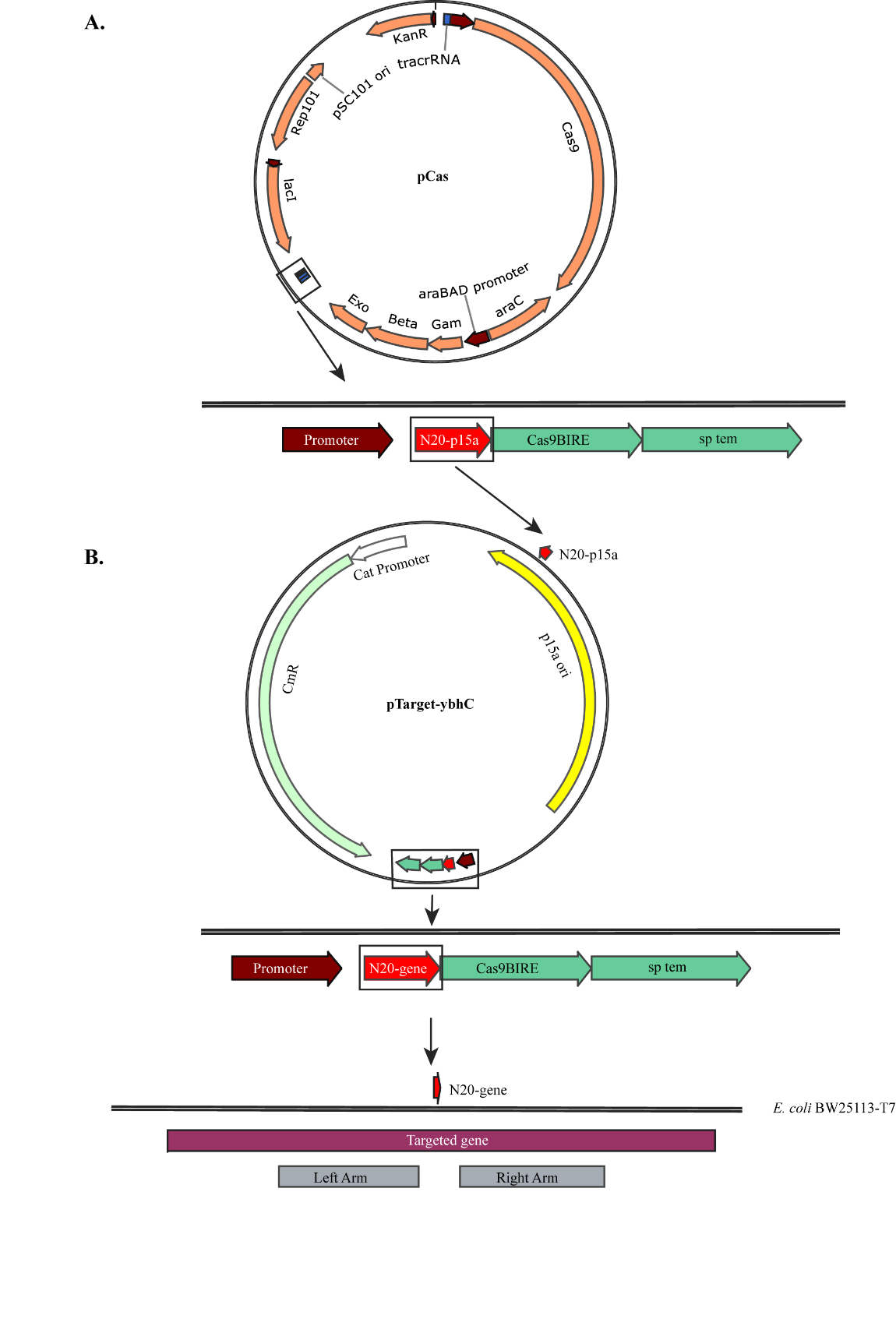


**Fig. S1**. Map of plasmids which were constructed for CRISPR. A) Map of pCas, which harbored the temperature sensitive oriR101 with repA101ts, kanamycin resistance gene, the λ-Red operon encoding Gam, Bet, and Exo proteins under the control of arabinose-inducible promoter ParaB, S. pyogenes-derived cas9 driven by endogenous promoters and sgRNA guided to ori-p15a which is under the control of lac operator. B) Map of pTarget-gene, which harbored Chloramphenicol resistance, ori-p15a and sgRNA guided to E. coli BW25113-T7 targeted gene.


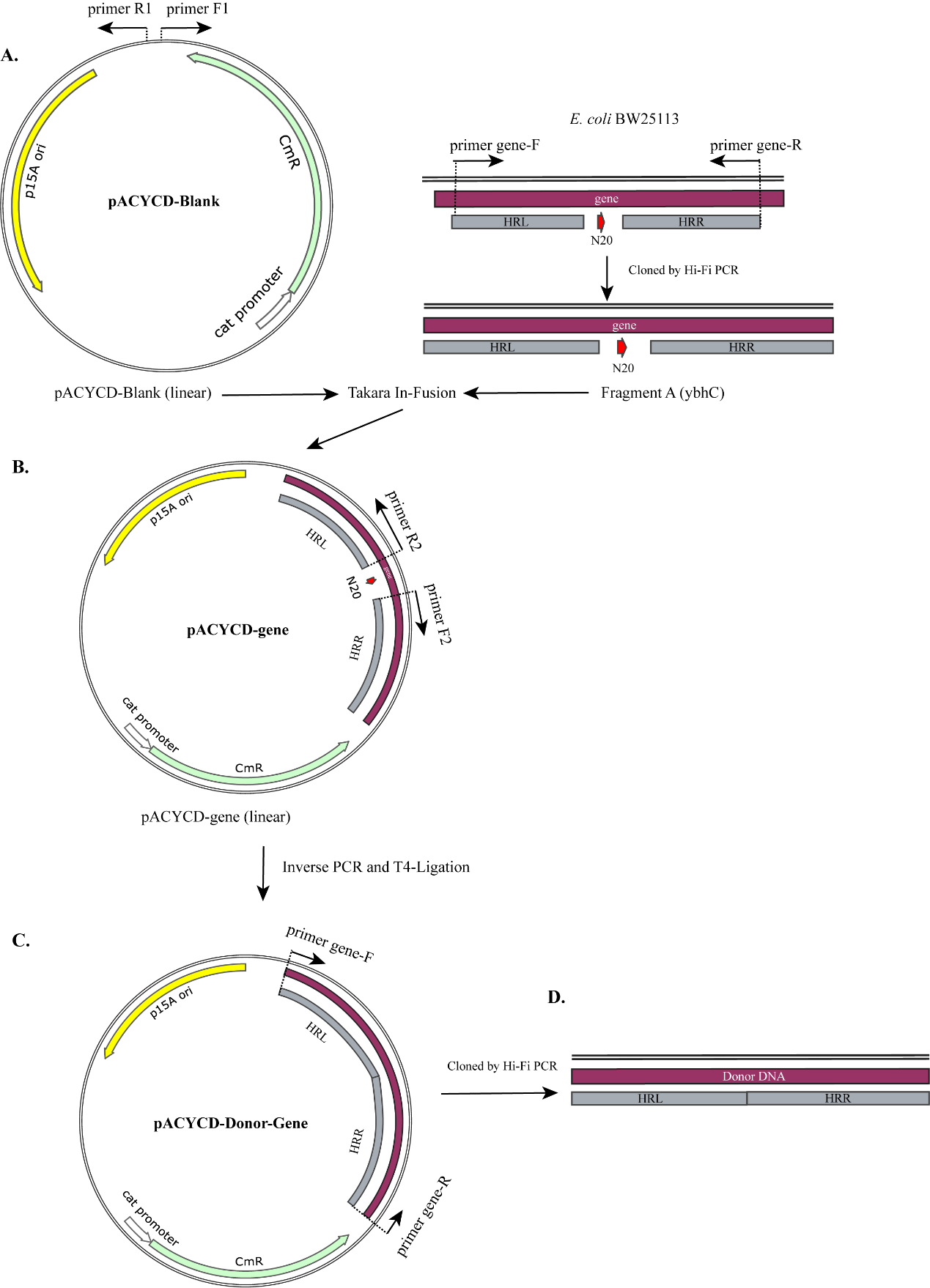


**Fig S2.** Construct of intermediate cloning vectors for preparing Donor DNA. (A) Fragment A cloned from BW25113-T7 was concatenated to pACYCD-Blank to assemble pACYCD-gene. (B) Reverse-PCR to constructed pACYCD-Donor. (C) The map of Donor DNA, which contains HRL and HRR.


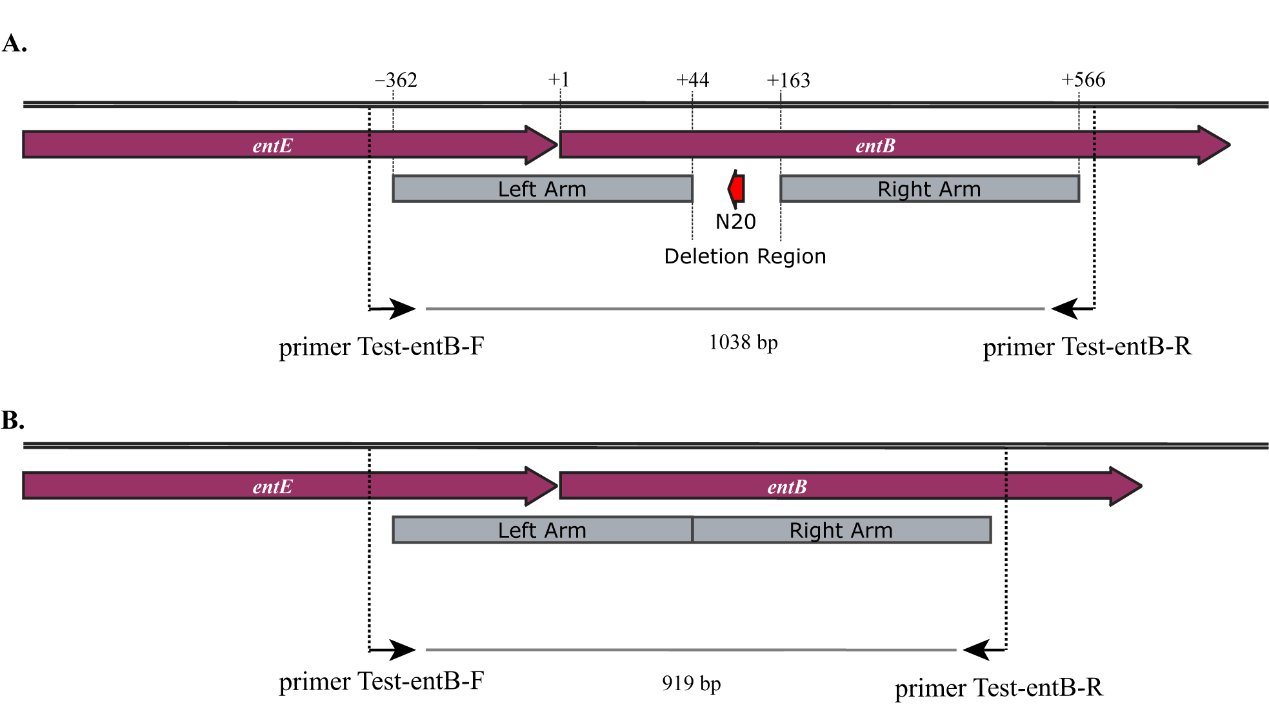


**Fig S3**. Gene map for targeted Cas9-mediated gene knock-out. (**A**) The knock-out site of *entB* in BW25113-T7. (**B**) map of *entB* knock-out in ideal condition and the location of PCR product (919 bp) for sequencing


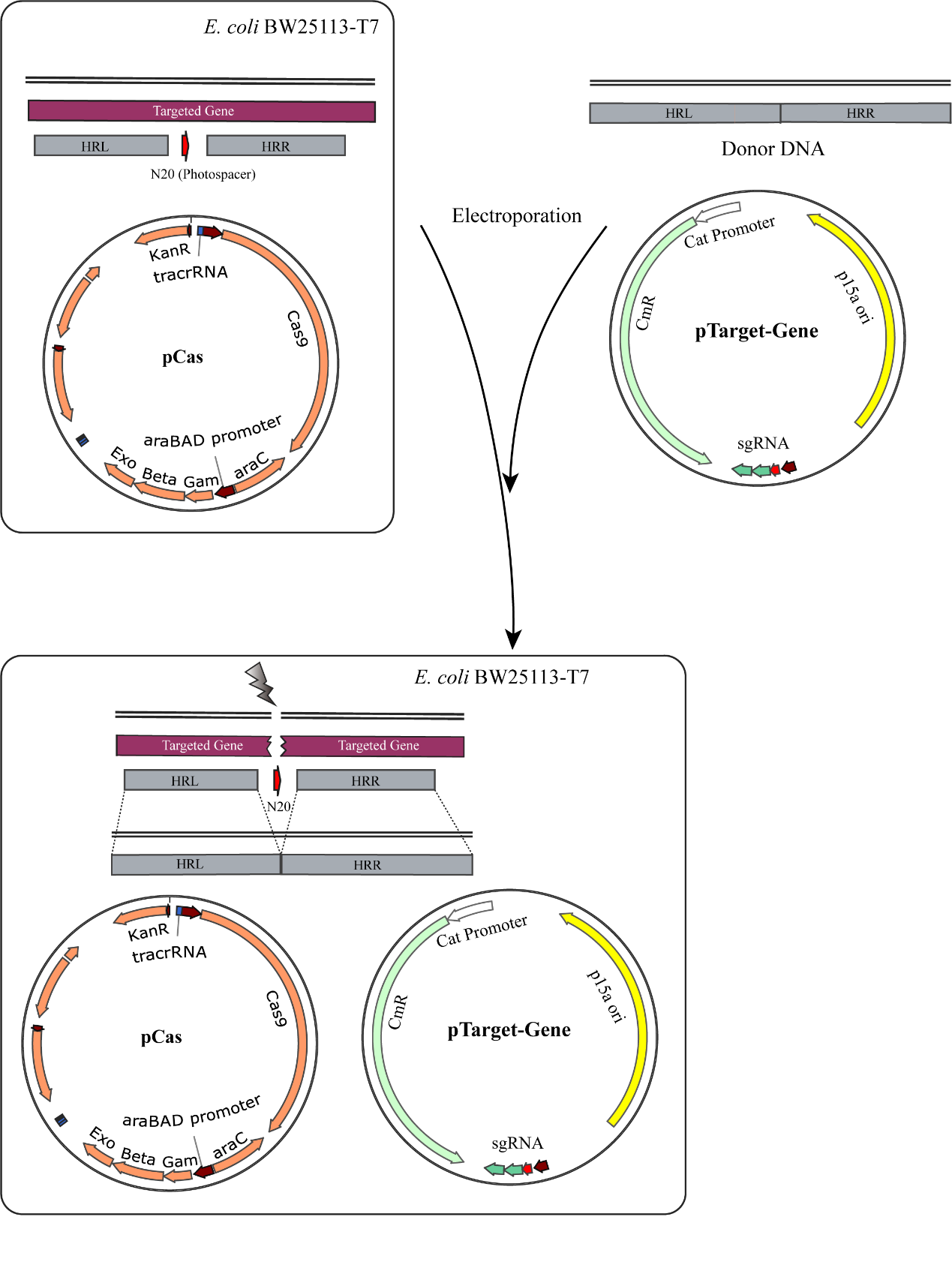


**Fig S4**. Schematic illustration of DSB induction and homologous recombination. After preparing competent cells, the pTarget-gene and Donor DNA which harbored homology arms (HRR and HRL) that targeted a chromosomal locus spanning the middle of targeted gene and the DSB site were electroporated into cells.

| \| **Primer** \| **Sequences (5'to3')** \| **bp** \| \| --- \| --- \| --- \| \| menA-R \| CTAGTATTTCTCCTCTTTCTCTAGAttatgctgcccactggctt \| 44 \| \| menA-F \| gaaggagatataccatatgactgaacaacaaattagccgaac \| 42 \| \| pET-R \| atggtatatctccttcttaaagttaaacaaaattattt \| 38 \| \| pET-F \| AGAGGAGAAATACTAGgagcaataactagcataaccccttg \| 41 \| \| Test-pEB-R \| ctttcagcaaaaaacccctcaag \| 23 \| \| Test-pEB-F \| ctcccttatgcgactcctgca \| 21 \| \| ubiE-R \| tgctagttattgctctcagaacttataaccacgatgcagc \| 40 \| \| ubiE-F \| GAGGAGAAATACTAGatggtggataagtcacaagaaacga \| 40 \| \| pET-ubiE-F \| gagcaataactagcataacccctt \| 24 \| \| VK-AC-R \| gctgctgcccatggtatatctcc \| 23 \| \| VK-AC-F \| aacctaggctgctgccacc \| 19 \| \| wrbA-F \| accatgggcagcagcatggctaaagttctggtgctt \| 36 \| \| wrbA-R \| gcagcagcctaggttttagccgttaagtttaactgccaga \| 40 \| \| qorB-F \| accatgggcagcagcatgatcgctattactggtgcc \| 36 \| \| qorB-R \| ggcagcagcctaggttctagttattaacattaaaaagatggct \| 43 \| \| Test-AC-R \| cggaagcagtgtgaccgtgt \| 20 \| \| menF-F \| accatgggcagcagcgtgcaatcacttactacggcg \| 36 \| \| menF-R \| cagcagcctaggttctattccatttgtaataaagtacgcagc \| 42 \| \| Seq-UC-F \| gaaaacctctgacacatgcagc \| 22 \| \| entB-F \| agagggttacatcaccgtgca \| 21 \| \| entB-R \| gacatcaaatgctcgtcacgg \| 21 \| \| UC-entB-F \| cgagcatttgatgtcctgtcgtgccagctgcatta \| 35 \| \| UC-entB-R \| gtgatgtaaccctctctgacgggcttgtctgctc \| 34 \| \| Donor-entB-R \| tgagactccggcagtgcgtaa \| 21 \| \| Donor-entB-F \| Aggtgatcgcgaatattgctgc \| 22 \| \| Test-entB-F \| tttgatgccaacggtttttactg \| 23 \| \| Test-entB-R \| gctggcagtaattcttcagtcat \| 23 \| \| N20-entB \| cgttgttaatccatgatatgactagtattatacctaggactga \| 43 \| \| pabC-F \| tttccacccttataaaaggtccgc \| 24 \| \| pabC-R \| cacttttttcatgactaattcgggcg \| 26 \| \| UC-pabC-F \| gtcatgaaaaaagtgctgtcgtgccagctgcatta \| 35 \| \| UC-pabC-R \| ttataagggtggaaactgacgggcttgtctgctc \| 34 \| \| Donor-pabC-F \| aacatctcaatcgtcttgagcaagt \| 25 \| \| Donor-pabC-R \| cggtccgctgttcaatgtgc \| 20 \| \| Test-pabC-F \| cggtggcactaatggttctttg \| 22 \| \| Test-pabC-R \| acccagtaccaccagcaataac \| 22 \| \| N20-pabC \| acgtaatcccctcgttacgcactagtattatacctaggactga \| 43 \| \| Seq-ACYCD \| ctcctccaagccagttacctc \| 21 \| |
| --- | --- | --- | --- | --- | --- | --- | --- | --- | --- | --- | --- | --- | --- | --- | --- | --- | --- | --- | --- | --- | --- | --- | --- | --- | --- | --- | --- | --- | --- | --- | --- | --- | --- | --- | --- | --- | --- | --- | --- | --- | --- | --- | --- | --- | --- | --- | --- | --- | --- | --- | --- | --- | --- | --- | --- | --- | --- | --- | --- | --- | --- | --- | --- | --- | --- | --- | --- | --- | --- | --- | --- | --- | --- | --- | --- | --- | --- | --- | --- | --- | --- | --- | --- | --- | --- | --- | --- | --- | --- | --- | --- | --- | --- | --- | --- | --- | --- | --- | --- | --- | --- | --- | --- | --- | --- | --- | --- | --- | --- | --- | --- | --- | --- | --- | --- | --- | --- |

**Table S1**. Primers used in this study.

**Table S2**. Growth rate of strain expressing various related genes in different medium.

| Strain | Growth Rate | Medium | Significance Summary |
| --- | --- | --- | --- |
| *E. coli* BW25113-T7 | 0.403 | LB |  |
| *E. coli* ΔB | 0.428 | LB | ns |
| *E. coli* ΔC | 0.393 | LB | ns |
| *E. coli* ΔBC | 0.406 | LB | ns |
|  |  |  |  |
| *E. coli* BW25113-T7 | 0.368 | M9-10G |  |
| *E. coli* ΔB | 0.376 | M9-10G | ns |
| *E. coli* ΔC | 0.382 | M9-10G | ns |
| *E. coli* ΔBC | 0.389 | M9-10G | ns |
